# Experiences Following a Sibling's Substance-Related Death: A Systematic Review

**DOI:** 10.1177/14550725251392045

**Published:** 2025-11-10

**Authors:** Heidi Jokipii, Anna Liisa Aho

**Affiliations:** 1 7840 Tampere University, RN, Tampere, Finland

**Keywords:** siblings, substance-related death, experience, grief, systematic review

## Abstract

**Background:** The death of a family member due to substance use may negatively affect family dynamics and the coping mechanisms of surviving family members, as the loved one may have already been perceived as lost during the period of substance use. Substance-related deaths are considered special due to their sudden nature and the circumstances surrounding the cause of death. The grief of siblings, in particular, is often overlooked, which highlights the importance of collecting comprehensive information about their experiences. **Aim:** The purpose of this systematic review is to describe siblings' experiences of losing a brother or sister to a substance-related death. The aim is to generate knowledge that enhances understanding of this topic and supports the development of appropriate and targeted support services. **Methods:** Nine articles were selected through the database search, and four additional articles were manually selected. The data from the initial search was analyzed using inductive content analysis. The results of the updated search were analyzed deductively on the basis of the previous results framework. **Findings:** The systematic review showed that siblings experienced being abandoned and carried the burden of loss. They also felt environmental insecurity and felt they will recover from grief. Support and assistance resources targeted at siblings are lacking. Attitudes and interactions of others have a significant impact on their grief experience.

## Introduction

The death of a sibling causes grief, which is a normal reaction to loss. Grief is a process in which one gradually adapts to the loss (De Stefano et al., 2021). Substance-related death can also cause feelings of anger, guilt, self-pity and relief because the bereaved may have anticipated the death in some way. All those feelings are complex but particularly guilt can complicate grief (Stroebe et al., 2024). Accepting reality and adapting to life without a loved one is important in grief (Stroebe & Schut, 1999). Thus, in light of the earlier statistics on substance use, it is important to gain information about the experiences of siblings after their sibling's death from substance use, in order to target the provision of the right kind of support. A study by Titlestad et al. (2021b) found that those who lost a loved one to substance-related death experienced the heaviest emotional burden and also received less support from their social environment than relatives of those who died in other ways.

A substance-related death is defined as a death primarily attributable to the use of illicit drugs, although alcohol and medicinal substances may also be involved in some cases (EUDA, 2025a). Substance-related death can also be defined as death by poisoning, death by accident, death by disease or suicide due to intoxication. In addition, substance-related death can be caused by violence and infectious disease in connection with substance use (Titlestad et al., 2021a). A substance-related death can also be called a drug-induced death or a drug-related death (EUDA, 2025a). The systematic review included twelve articles on drug-related deaths and one article on a death involving both alcohol and drugs. This systematic review uses the term substance-related death, which refers to all deaths where a sibling has died due to drugs or alcohol.

In the European Union, it is estimated that almost 7500 people died of a substance use in 2023 and it appears that opioids combined with other substances are the most common reason in substance-related deaths (EUDA, 2025b). The researchers in the present study are from Finland, where there has been much discussion about substance-related deaths. In 2023, 310 people died from drugs in Finland and every third person who died was aged under 25 years. The majority of substance-related deaths were accidental poisonings, which usually involve the simultaneous use of several substances. In total, 30 substance-related deaths were suicides with drugs or drugs classified as narcotics. Finland saw its highest number of substance-related deaths that year (Tilastokeskus, 2024). Yet, as substance-related deaths increase, the number of siblings who die from substance-related illnesses also increases in our society.

A substance-related death is difficult because of the stigma associated with it. The subject is shameful and associated with prejudice, secrecy and denial (Da Silva et al., 2007; THL, 2025). Because of a social stigma associated with the cause of death, grief can be worse (Funk et al., 2018). Losing a loved one is a painful experience, causing sadness and anxiety (Treml et al., 2022). According to Shear and Solomon (2015), some forms of grief are more difficult than others. Grief may become further complicated if there are traumatic circumstances surrounding the death, which are usually encountered in the case of substance-related deaths (Shear & Solomon, 2015).

In grief research, siblings have been described as forgotten mourners (McCoyd & Walter, 2016). Siblings as the bereaved are the least studied familial relationship (Rostila et al., 2012). This systematic literature review brings together all the former research findings from the sibling perspective that further research can be targeted to areas where little research is available.

The purpose of this systematic review is to describe siblings’ experiences after losing a brother or sister to a substance-related death. In this systematic review, sibling refers to all those siblings who have lost a sibling through substance-related death. The age of the sibling is not limited in the review.

The aim is to generate knowledge that enhances understanding of this topic. The information will help to better understand the grief process and experiences of siblings. This also helps in targeting the provision of the right kind of support to siblings who have experienced a substance-related death. The research question of the systematic review is what types of experiences siblings have regarding the substance-related death of their brother or sister.

## Methods

### Data Sources and Search Strategy

In a systematic review, the aim is to systematically search for, evaluate and comprehensively combine research evidence on a specific topic. The reporting of the systematic review is transparent and the review is repeatable (Grant & Booth, 2009) and it identifies topics where research is still needed (Petticrew & Roberts, 2008).

In this systematic review, the search for information was carried out by the first researcher (HJ). The search terms of this systematic review were adapted from the central concepts of the research question, synonyms of the concepts and parallel terms (Table 1). The search words were separated with an asterisk symbol (*) to make it possible to use alternative spellings of the search word.

**Table 1.** Databases and Search Terms Used.

Database	Search terms
CINAHL (EBSCO) Medline (EBSCO) PsycInfo (Ovid)	**Sibling ** Sibling* OR Brother* OR Sister **Substance abuse ** Drug* OR Alcohol* OR “Substance Abuse*nbsp; **Death ** Death OR Overdose OR “Alcoholic Intoxication ” **Experience ** Experienc* OR View* OR Interpretation* OR Thought* OR Attitude* OR Emotion*

The search was conducted using the CINAHL (http://ebsco.com/products/research-databases/cinahl-database), Medline (https://www.nlm.nih.gov/medline/medline_home.html) and PsycInfo (https://www.apa.org/pubs/databases/psycinfo) databases. The same search phrase formed by free words was used in all databases. The initial search was carried out in August 2023, and an academic IT specialist was used to plan the search terms and strategy.

In the databases, search terms were combined using the Boolean operators AND, OR and NOT. Articles published before 2013 were excluded from the search results. After this limit was imposed, 25 search returns remained in CINAHL, 60 in Medline and 35 in PsycInfo. Due to the narrow search result, a broader search was tried starting in 2008, although this did not produce more returns. Only peer-reviewed articles were considered for review, culminating in 23 search results from CINAHL, 55 from Medline, and 28 from PsycInfo.

All 106 search results were transferred to the reference management program Zotero (https://www.zotero.org) and 32 duplicates were removed. In total, 74 articles were read at the abstract level by the first researcher. Based on the abstract, 64 articles that did not answer the research question were rejected. The articles acceptance criteria stipulated a scientific peer-reviewed original article which focused on “the sibling's perspective” and related to “a sibling's substance-related death”. All deaths involving drugs, medications or alcohol were accepted for the literature review. Exclusion criteria were studies with only the perspective of another family member or other causes of death. However, the researchers included all of those studies that included siblings together with other family members as participants. Consequently, the systematic search yielded seven research articles for review. Those articles were selected by the first researcher (HJ).

**Figure 1.**
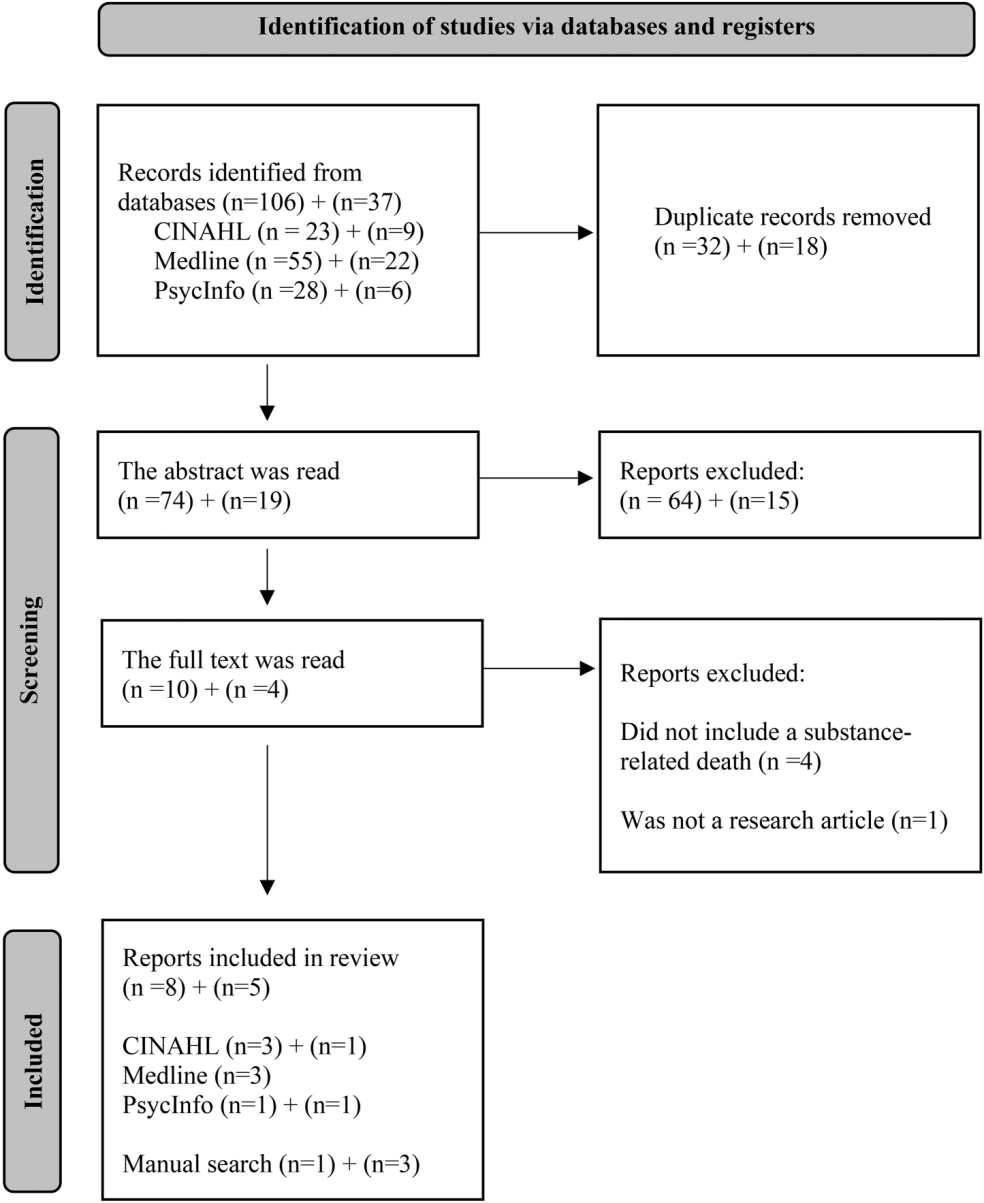
Description of the Application Process Using the Prisma Diagram.

The updated database search was conducted in July 2025. The updated search returned 56 more results from CINAHL, 127 from Medline and 91 from PsycInfo. After applying the inclusion and exclusion criteria and removing 18 duplicates, 19 articles remained. Those articles were read at the abstract level. The update search yielded two more articles to this systematic review. The PRISMA flow diagram shows the results of the initial search first and then the results of the updated search (Figure 1).

A manual search was also performed on Google Scholar (https://scholar.google.com) with the search term (Sibling* AND “Drug Related Death*”), which yielded one more article for inclusion in the systematic review in August 2023 and three more articles in September 2025. There was also backward citation searching made from the reference lists of articles manually selected by the researcher but no further sources were identified. In total, 13 articles were selected for the systematic review in September 2025 (Table 2).

**Table 2.** Articles Selected for the Review.

Authors, year, country	The purpose of the study	Data and research methods	Results	Quality assessment
Dyregrov & Selseng, 2022, Norway	To describe the stigmatization of someone who has lost a loved one in a drug-related death, and its appearance and content	The data is a national cross-sectional study. A mixed methods study. Unstructured questionnaire, n = 106. 31% of respondents (n = 31) were siblings. Thematic analysis	Stigma related to substance use makes the grieving process and receiving support difficult. The siblings who lost their sibling in a substance-related death had heard numerous negative comments about the deceased, which were difficult to deal with	5/6, 9/10
Dyregrov et al., 2022, Norway	To describe the experiences of grieving siblings with informal support after a sibling's substance-related death	The data is a national cross-sectional study. Qualitative research. Semi-structured interview, n = 10 siblings. Reflective thematic analysis	The grieving siblings had not received peer support. Peer support would have been perceived as useful. In promoting the support of social networks, the most important factors are openness and mutual closeness	10/10
Fjær & Dyregrov, 2021, Norway	To develop support services for the bereaved after a substance-related death and to make their voices heard in the political debate	The data comes from a national cross-sectional study. Unstructured questionnaire, n = 83. Of the respondents, 31% are siblings. Thematic analysis	Those who have lost a family member or friend to a drug-related death receive support and help later compared to other grieving groups. Wide-ranging, routine, respectful and competent help should be available	8/10
Meen et al., 2025, Norway	To develop an understanding of how such meaning-making stories are socially and culturally embedded and how they influence the coping and adjustment of siblings	The data is a national cross-sectional study. Qualitative research. Semi-structured interviews, n = 14 siblings. Discursive Psychology	Four groups of sense-making stories about drug-related deaths were found: ‘death as the only outcome,’ ‘death caused by difficulties in the family,’ ‘death caused by lack of public help’, and ‘stories of uncertainty and doubt’	10/10
Meen et al., 2024a, Norway	To investigate how social categories work and intersect in siblings bereaved by drug-related deaths’ stories about their relationships to their deceased brother or sister	The data is a national cross-sectional study. Qualitative research. Semi-structured interviews, n = 14 siblings. An intersectional analysis	Categorization of the deceased siblings as “addicts” constructs a troubled position Normative conceptions of addiction and DRDs produce troubled subject positions Intersections of categories can add further complexities of remorse and self-blame for the bereaved siblings	10/10
Meen et al., 2024b, Norway	To investigate how bereaved siblings of drug-related deaths engaged with grief-related discourses and how they defined their roles and identities in the context of loss	The data is a national cross-sectional study. Qualitative research. In-depth interviews, n = 10 siblings. Discursive psychology	There are four distinct interpretative repertoires in how siblings spoke about grief: grief as visibly expressed, grief as structured in a hierarchy, grief as a process to be managed, and grief as a response to death. These repertoires reflect the diverse ways in which siblings make sense of their bereavement and construct meaning from their experiences	10/10
Lambert et al., 2021, Ireland	To examine how complicated grief affects family dynamics after the drug-related death of a loved one	Qualitative research. Six focus group interviews and one semi structured interview, n = 17 family members. Of the respondents, 6 are siblings. Reflexive thematic analysis	There are three central themes: changes in relationships, emotional complexity, and adapting to life after loss. The results highlight that individuals in this group face significant challenges in coping with grief, particularly due to family disruption, limited access to support, and the impact of stigma	10/10
Lindeman et al., 2023, Norway	To describe how siblings gather meanings from their family relationships when a sibling has a substance abuse problem	The data comes from a national cross-sectional study. Qualitative research. Semi-structured interview, n = 14 siblings, retrospective focus. Narrative thematic analysis	Living with a sibling who uses substances is complicated and multifaceted. Siblings’ lives are affected by their sister's or brother's problems, which is why siblings should also receive support and be involved in treatment practices even before the sibling's substance-related death	9/10
Løberg et al., 2022, Norway	To describe the burden of siblings before the death of their sibling	The data comes from a national cross-sectional study. Qualitative research. Semi-structured interview, n = 14 siblings, retrospective focus. Thematic analysis	The siblings offered their substance-using sibling emotional support, social inclusion and practical support	9/10
O’Callaghan et al., 2022, Ireland	To investigate how families cope and grow after losing a loved one to a drug-related death	Qualitative research. Six focus group interviews and one semi structured interview, n = 17 family members. Of the respondents, 6 are siblings. Reflexive thematic analysis	The analysis revealed themes of resilience and personal development. Families may experience post-traumatic growth as they start to accept their loss and receive support throughout their grieving journey	10/10
Perrin, 2023, Australia	To present information on topics related to drugs and alcohol, the study of death and family therapy theory, promoting the understanding of these perspectives and showing how different perspectives bring out different understandings	The material was based on the qualitative material produced for the dissertation. Semi-structured interview, n = 21 siblings. The article focused on the story of one interviewee. A phenomenological approach	When human experience is viewed from different perspectives, a deeper and more multi-level understanding of the same experience is obtained Ignorance and incomprehension of the seriousness of drug use made the sibling's substance-related death an unexpected death. When grieving the death of a sibling, the importance of close people was emphasized. Being open about the cause of death helped when dealing with grief	8/10
Templeton et al., 2016, England	To describe the experiences and needs of grieving people	Qualitative research. Semi-structured interview. Interviews n = 100, interviewees n = 106 (56 parents, 21 children, 13 spouses, 13 siblings, 6 friends and 3 nieces) A phenomenological approach	Living with substance use, experiencing subsequent death, and the reactions of professionals and others can rob grief and negatively affect grieving and seeking support	9/10
Titlestad & Dyregrov, 2022, Norway	To assess the prevalence of symptoms of prolonged grief in grieving family members after a substance-related death, to identify predictive factors and to assess whether the symptoms decrease over time	The data is from a cross-sectional study A cross-sectional study Structured questionnaire, n = 234 (93 parents, 78 siblings, 24 children, 39 other family members)	The symptoms of prolonged grief were highest among those who had lost a family member 1–2 years ago Symptoms of prolonged grief decreased over time, although those who lost a family member 6–8 years ago had more symptoms of prolonged grief than the group who lost a family member 4–6 years ago	7/8

### Quality of Included Studies

A quality assessment of the selected research articles was carried out to strengthen the trustworthiness of the review (Petticrew & Roberts, 2008). In this systematic review, the two researchers (HJ and ALA) first evaluated the quality of the studies independently and then compared their evaluations. The evaluation criteria of the Joanna Briggs Institute were used (JBI, 2025). Eleven qualitative studies were selected for the systematic review, for which the qualitative research checklist was used (Hotus, 2018a). The mixed methods study used a checklist for both qualitative research and cross-sectional research (Hotus, 2019). The checklist for the cross-sectional study contains eight evaluation criteria, two of which were not applicable to the study being evaluated. In the cross-sectional study selected for the systematic review, the cross-sectional study evaluation criteria were used for quality assessment (Hotus, 2019) (Table 2).

### Data Analysis

The material of the systematic review was analyzed using inductive content analysis and deductive content analysis was used to analyze five articles selected through an update search. In inductive content analysis, it is important to formulate the research questions as well as the purpose and aim of the research in such a way that answers can be obtained from the material through the analysis of the content (Elo et al., 2022). The analysis of the data started by extracting original expressions corresponding to the research question from the results sections of the articles selected for review. If the study included other family members, only those sections of the results section were taken for analysis where it was certain that the results were directed at siblings. The articles selected for the study were in English. Table 3 shows three examples of reducing the original expression.

**Table 3.** Reduction of the Original Expression.

Original expression	Reduction
“… when they needed it (help) the most — it was then difficult to ask for more” (Dyregrov et al., 2022)	Difficulty asking for more help (Dyregrov et al., 2022)
“… because people are run down, few will actively seek psychological help” (Fjær & Dyregrov, 2021)	Few manage to seek psychological help (Fjær & Dyregrov, 2021)
“… how much harder things are when support is unavailable or poorly joined up” (Templeton et al., 2016)	Due to the lack of support, things were more difficult (7) Due to poorly organized support, things were more difficult (Templeton et al., 2016)

Reduced expressions (*n* = 146) were compared and combined to find similarities in siblings’ experiences. Expressions that meant the same thing were collected together, so that emerging subcategories could be formed (Elo et al., 2022). By grouping the data, a total of 29 emerging subcategories were formed. Emerging categories were formed by comparing subcategories and combining subcategories with the same content. The categories were named in such a way that they well describe their content.

### Description of the Included Studies

Of the articles selected for the systematic review (*N* = 13), nine originated from Norway (Dyregrov & Selseng, 2022; Dyregrov et al., 2022; Fjær & Dyregrov, 2021; Lindeman et al., 2023; Løberg et al., 2022; Titlestad & Dyregrov, 2022; Meen et al., 2025; Meen et al., 2024a; Meen et al., 2024b), two from Ireland (Lambert et al., 2021; O’Callaghan et al., 2022), one from Australia (Perrin, 2023) and one from England (Templeton et al., 2016). The materials of the Norwegian studies come from the END project, the main goal of which is to improve the life situation of those left to mourn a substance-related death (The END project, 2023).

Eleven qualitative studies were selected for the systematic review (Dyregrov et al., 2022; Fjær & Dyregrov, 2021; Lambert et al., 2021; Lindeman et al., 2023; Løberg et al., 2022; Perrin, 2023; Templeton et al., 2016; Meen et al., 2025; Meen et al., 2024a; Meen et al., 2024b; O’Callaghan et al., 2022), one mixed methods study (Dyregrov & Selseng, 2022) and one cross-sectional study (Titlestad & Dyregrov, 2022). In seven studies, the interviewees were all siblings of persons with a substance use disorder (Dyregrov et al., 2022; Lindeman et al., 2023; Løberg et al., 2022; Perrin, 2023; Meen et al., 2025; Meen et al., 2024a; Meen et al., 2024b). In five studies, about one-third of the interviewees were siblings (Dyregrov & Selseng, 2022; Fjær & Dyregrov, 2021; Lambert et al., 2021; O’Callaghan et al., 2022; Titlestad & Dyregrov, 2022). In one study, 13 siblings were interviewed out of a total of 100 participants (Templeton et al., 2016).

Articles where respondents included people other than siblings the respondents’ ages varied between 21 and 80 years (Dyregrov & Selseng, 2022), 22 and 75 years (Templeton et al., 2016), and 23 and 71 years (Fjær & Dyregrov, 2021). The age of the responded siblings varied between 23‒61 years (Løberg et al., 2022), and 30‒61 years (Dyregrov et al., 2022; Lindeman et al., 2023; Meen et al., 2025; Meen et al., 2024a; Meen et al., 2024b). The ages of the deceased ranged from 16 to 84 years (Templeton et al., 2016), 17 to 50 years (Lindeman et al., 2023), 18 to 68 years (Fjær & Dyregrov, 2021), 19 to 46 years (Lambert et al., 2021; O’Callaghan et al., 2022), and 24 to 41 years (Dyregrov & Selseng, 2022). A minimum of 3 months had passed since death (Fjær & Dyregrov, 2021; Løberg et al., 2022), with a maximum of 30 years (Lindeman et al., 2023; Løberg et al., 2022; Meen et al., 2025; Meen et al., 2024a) (Table 2).

## Results

Using inductive content analysis, three main categories and eight subcategories were formed from the siblings’ experiences. After their sibling's substance-related death, the siblings experienced loneliness, comprehensive grief, dysfunctional family dynamics and stigmatization. In addition, the siblings experienced an increased importance of loved ones, acceptance of loss, self-esteem and a need for increased understanding (Table 4).

**Table 4.** The Experiences of Siblings After the Substance-related Death of Their Sibling.

Main theme	Emerging category	Emerging subcategory
Being rejected and bearing the burden of loss	Experiencing loneliness, The comprehensive grief	Being left alone, Challenges to get help, Experiencing the incomprehension of others, Experiencing powerlessness, Losing, Experiencing hopelessness, Experiencing the unexpectedness of death, Experiencing sadness
Environmental insecurity	Dysfunctional family dynamics experiencing, Stigmatization	Taking responsibility for the family, Experiencing conflicts within the family, Hiding feelings, Experiencing accusations, Experiencing shame, Experiencing inappropriate treatment, Blaming the deceased, Feeling worthless, Self-blaming
Recovering from grief	The emphasized meaning of loved ones, Accepting loss, Valuing oneself, The need to increase understanding	Experiencing the importance of support from loved ones, Convergence of family relationships, Experiencing comfort, Experiencing the importance of practical help, Experiencing openness, Experiencing relief, Experiencing peace, Experiencing a valuable encounter, Distancing and setting boundaries, Taking care of oneself, The need for answers about death, The need to understand substance addiction

### Being Rrejected and Bearing the Burden of Loss

The siblings’ experience of being abandoned and bearing the burden of loss involved experiencing loneliness and total sadness. Siblings’ loneliness was related to their experiences of being left alone and it was especially felt due to the lack of peer support and the lack of help offered overall (Dyregrov et al., 2022; Lambert et al., 2021; Meen et al., 2024b). The lack of help was perceived to be connected to the substance-related death (Fjær & Dyregrov, 2021; Lambert et al., 2021) and caused disappointment for siblings (Dyregrov et al., 2022). One of the challenges of getting help was that you had to seek help yourself and it was difficult to find because of the grief and shock. Furthermore, the siblings didn't always know what kind of help they needed or where to find help, and only some of the siblings sought help after their sibling's substance-related death (Fjær & Dyregrov, 2021).

Other people's incomprehension was related to the siblings’ experiences of not recognizing the family's need for help and the poor quality of the support provided (Meen et al., 2024b; Templeton et al., 2016). In addition, the siblings felt that support was provided inefficiently and that the support provided ended too early (Dyregrov et al., 2022). Especially, people who knew the dead sibling only as a person with a substance use disorder did not offer support (Dyregrov & Selseng, 2022). Siblings also experienced powerlessness due to lack of support (Templeton et al., 2016), difficulty in asking for help (Dyregrov et al., 2022; Meen et al., 2024b) and feeling they did not have the strength to contact anyone (Fjær & Dyregrov, 2021).

The comprehensive grief experienced by the siblings was related to losing a sibling (Fjær & Dyregrov, 2021; Lindeman et al., 2023; Meen et al., 2024a; Templeton et al., 2016). The siblings experienced grief and the loss of their sibling even before their death, due to their substance use (O’Callaghan et al., 2022; Lambert et al., 2021; Meen et al., 2024b). The siblings felt it was important that their sibling not be remembered only as a person with substance use disorder (Meen et al., 2024a). The hopelessness experienced by the siblings was related to their sense of insignificance (Fjær & Dyregrov, 2021) and the loss of hope for their sibling's recovery (Lambert et al., 2021; Lindeman et al., 2023; O’Callaghan et al., 2022). The experience of the unexpectedness of death was influenced by a lack of understanding of the sibling's substance use (Perrin, 2023) and the seriousness of the whole situation (Lindeman et al., 2023).

Sadness included the siblings’ experiences and feelings towards their deceased sibling (Lambert et al., 2021; Meen et al., 2024b; Templeton et al., 2016). Sadness was aggravated by things experienced before the death, and also the nature of the death (O’Callaghan et al., 2022; Templeton et al., 2016). Titlestad and Dyregrov (2022) found that 26 percent of mourners suffered from strong symptoms of prolonged grief. However, grief was strong for the whole family (Lindeman et al., 2023) and felt to be strongest to each person in the family 1–2 years after the death of the family member (Titlestad & Dyregrov, 2022). Intense grief was also perceived to be an obstacle to communication (Dyregrov et al., 2022).

### Environmental Insecurity

Experiencing the insecurity of the environment included experiencing dysfunctional family dynamics and being stigmatized. Dysfunctional family dynamics were reflected in how siblings took on caregiving roles for their substance-using sibling prior to the death (Løberg et al., 2022). Siblings felt responsible for their sibling by providing financial and emotional support, as well as practical help (Løberg et al., 2022). Siblings also act as their siblings’ psychologists (Lindeman et al., 2023) and family balance maintainers (Løberg et al., 2022; Lambert et al., 2021; Meen et al., 2024b). Taking responsibility involved the siblings’ attempt to be the perfect child for their parents and a desire to please their parents in the role of helper (Lindeman et al., 2023; O’Callaghan et al., 2022). Siblings felt further responsible as supporters of the whole family's mental well-being (Løberg et al., 2022).

Experiences of family disagreements (Dyregrov et al., 2022; Lambert et al., 2021) and complex family history (Lindeman et al., 2023) were associated with experiencing conflicts within the family. Bad family relations were even more intensified because feelings arose that had not been talked about before (Dyregrov et al., 2022). The siblings’ feelings were conflicting because the sibling who used substances received all the attention within the family (Løberg et al., 2022). Siblings understood their parents, but being ignored was still perceived as hurtful (Lindeman et al., 2023). Siblings understood their parents’ grief (Lindeman et al., 2023) and avoided emphasizing their own successes so that their sibling's failures would not come to light (Løberg et al., 2022). Afterwards, the sibling often wished that someone had told them not to take on all that responsibility for the family (Lindeman et al., 2023).

Hiding feelings involved hiding feelings from others. The siblings did not show their own needs and their feeling of sadness was minimized (Meen et al., 2024b). The siblings also did not want to be a burden to others, which is why they kept their grief hidden from view (Lambert et al., 2021). Grief also caused exhaustion, which made it difficult to show emotions (Dyregrov et al., 2022).

Experiencing accusations relating to a sibling's substance use disorder and death made the sibling feel like a criminal (Templeton et al., 2016). The rest of the family placed expectations on the siblings, especially in regard to solving and helping their substance-using sibling's problems and acting as their sibling's counselor (Lindeman et al., 2023). A sibling could even be blamed for their sibling's death (Dyregrov et al., 2022).

Stigma was associated with experiences of shame. Experiencing shame due to the cause of death prevented siblings receiving support from their social network (Dyregrov et al., 2022; Meen et al., 2024b). Shame made the sibling hide the truth (Meen et al., 2024a; Templeton et al., 2016) and avoid talking about the sibling's death and its cause (Dyregrov et al., 2022). Due to the cause of the death, the siblings doubted their right to mourn their dead sibling (Templeton et al., 2016). The stigmatizing attitudes experienced by the siblings were associated with experiencing inappropriate treatment (Dyregrov & Selseng, 2022; Meen et al., 2024b). In particular, the siblings had bad experiences when encountering the police and their treatment was perceived as inappropriate and disturbing. Stigmatizing attitudes were experienced from the media, relatives, coworkers, neighbors and friends (Templeton et al., 2016). However, mistreatment included stereotypical labeling and dehumanization (Dyregrov & Selseng, 2022). Overall, the siblings would have liked to be met without stigmatizing attitudes (Fjær & Dyregrov, 2021). The substance-related death of their sibling was commented on bluntly, disrespectfully and maliciously, and the inappropriate comments of others added to their perceived sadness (Dyregrov & Selseng, 2022).

Blaming the deceased was accompanied by messages from other people about self-inflicted death, the only real option and the best solution due to the use of substances. The substance use disorder was assumed to be self-selected and intentional, and the death was said to have spared the sibling the burden of caring for a person with a substance use disorder (Dyregrov & Selseng, 2022; Lambert et al., 2021; Meen et al., 2024b).

Feeling worthless was related to the siblings’ experience that, due to a substance-related death, other mourners were treated differently (Fjær & Dyregrov, 2021; Lambert et al., 2021; Meen et al., 2024b). Siblings tried to protect the memory of the deceased (Templeton et al., 2016), but, at the same time, felt worthless (Dyregrov et al., 2022). Rude comments from other people were difficult for the siblings to deal with, and they left painful scars for years and prevented them from keeping good memories (Dyregrov & Selseng, 2022).

Self-blame appeared in the form of the siblings’ experience of guilt (Meen et al., 2024b), especially that their sibling had died alone (Templeton et al., 2016). Guilt caused anxiety (Fjær & Dyregrov, 2021; Meen et al., 2024b), increased low self-esteem (Lindeman et al., 2023) and negatively affected sibling relationships (Lindeman et al., 2023). Siblings also experienced guilt due to failed attempts to help (Lambert et al., 2021; Lindeman et al., 2023) and sometimes blamed themselves for their sibling's death (Titlestad & Dyregrov, 2022).

### Recovering from Grief

Recovery from grief included emphasizing the meaning of loved ones, accepting the loss, valuing oneself and the need for increased understanding. The siblings’ experience of the importance of loved ones was reflected in how they valued the support received from friends and family (Dyregrov et al., 2022; Meen et al., 2024b). Support from friends was perceived as important (Meen et al., 2024b; Perrin, 2023) and especially from friends who had also lost their siblings (Dyregrov et al., 2022; Lambert et al., 2021; O’Callaghan et al., 2022). Convergence of family relationships was related to family members supporting each other and becoming closer (Lindeman et al., 2023; Meen et al., 2025). The importance of family and the connection with other siblings were both emphasized (Perrin, 2023) and stable family relationships guaranteed spontaneous support (Dyregrov et al., 2022).

The experience of comfort was increased by the dead sibling's friends’ memories of the sibling and their sibling's last days (Dyregrov et al., 2022), which the siblings appreciated. The funeral was perceived as an occasion where it came out how the deceased sibling was loved (Templeton et al., 2016) and how he/she meant something to others (Dyregrov et al., 2022). Creative ways of remembering their sibling brought comfort (Templeton et al., 2016). Even after years had passed, mementos of the sibling were kept and the siblings had the feeling that the deceased sibling was still present in their lives (Perrin, 2023).

Experiencing the importance of practical help was accompanied by an appreciation for small actions. The siblings felt immediate support was important. Versatile practical support brought comfort. Practical help included both listening to siblings and bringing food. Empathy was shown by bringing flowers (Dyregrov et al., 2022). Experiencing openness was associated with honesty about the cause of death (Templeton et al., 2016) and telling the truth about the death of a sibling (Dyregrov et al., 2022). Openness and closeness were perceived as important in receiving support (Dyregrov et al., 2022). Openness communicated to others that the sibling's substance use was not something that should be kept quiet (Dyregrov et al., 2022). Also, a public memorial service was felt to help with grief (Perrin, 2023).

Siblings’ acceptance of the loss was associated with a sense of relief, even though the death of a sibling was simultaneously experienced as deeply saddening (Lambert et al., 2021; Lindeman et al., 2023; Meen et al., 2024b; O’Callaghan et al., 2022). Complex and intense emotions were common (Templeton et al., 2016). The sibling experienced relief for themself and their sibling (Templeton et al., 2016). However, feeling relief also caused feelings of guilt (Titlestad & Dyregrov, 2022). The opportunity to be present at the time of the sibling's death helped them experience peace and seeing the body gave them the opportunity to say goodbye. A sympathetic, supportive and respectful encounter with the authorities contributed to a meaningful experience. Particularly, a small number of siblings did not experience stigmatization, and this was helped by the non-judgmental attitude of the professionals they encountered (Templeton et al., 2016).

Distancing themselves and setting boundaries related to valuing oneself were central in the life of a sibling (Lambert et al., 2021; Løberg et al., 2022). The siblings felt that setting boundaries was necessary to secure their own quality of life and health (Lambert et al., 2021; Løberg et al., 2022). The desire to distance themselves (Lindeman et al., 2023) or to be alone (Dyregrov et al., 2022) helped siblings protect themselves (Lindeman et al., 2023) and taking care of oneself involved the need to control one's own emotions (Templeton et al., 2016), to be strong and to trust only oneself (Dyregrov et al., 2022).

The need to get answers about death was related to the siblings’ desire to know about death and the cause of death (Fjær & Dyregrov, 2021; Lambert et al., 2021). The siblings wanted to gain an understanding of the time before and after death (Fjær & Dyregrov, 2021). Siblings also had a need to talk about death more than their grief (Dyregrov et al., 2022) and, in some cases, the knowledge could add additional pain (Templeton et al., 2016). The need to understand drug addiction was connected to the siblings’ desire to seek more information about drugs and addiction (Meen et al., 2024a; Templeton et al., 2016) and it was important for siblings to gain an understanding of their sibling's substance use disorder (Lindeman et al., 2023) and understand that addiction is a disease (Meen et al., 2024a).

## Discussion

The results show a wide range of siblings’ experiences, from comprehensive grief to the acceptance of a sibling's death. Grief should be treated properly, and exercise, talking and seeking help all play an essential role (Meen et al., 2024b). Crisis help is therefore of paramount importance when facing grief, and, at a later stage, professional help and healthcare services should be easily available. However, overall, the support received by the siblings was poor, and there was no continuity of support after the end of the acute phase (Fjær & Dyregrov, 2021). Sibling death in childhood increases risk of psychiatric treatment in adulthood. The risk is high if the death has been sudden or traumatic. Therefore, it is important to recognize and deal with sibling grief as part of the family's grieving process (Rostila et al., 2019).

According to the results, the siblings found it difficult to monitor their sibling's substance use even when he/she was alive. Even when any hope of stopping the sibling's substance use was lost, the sibling's death was still unexpected due to a lack of understanding of the seriousness of substance use. In Finland, attitudes and opinions regarding the use of substances have changed in a more accepting direction, and, according to data collected by the Institute of Health and Welfare in 2022, Finns’ drug experiments and drug use have consistently become more common (THL, 2024). This is why increasing education about substance use disorder is important because it helps individuals gain the skills needed to take care of their own well-being and the well-being of others. Particularly, it helps in creating truthful expectations about the consequences of substance use (EHYT ry, 2025).

The findings showed that, after a sibling's death, dysfunctional family dynamics became more prominent and the siblings felt that they were responsible for the rest of the family. Funk et al. (2018) studied siblings who were left alone with their grief and felt that their grief was being ignored, especially if their parents withdrew. Parents are seen as being more entitled to mourn than those who have lost a sibling (Funk et al., 2018). The death of the sibling increased the visible perception of differences in the family, and, if you weren't used to talking about difficult things, you kept quiet about them (Dyregrov et al., 2022). The family members thus did not receive support from each other, which would be crucial in a crisis situation (Weaver et al., 2023). The siblings minimized their own grief because they didn't want others to see them as a burden (Dyregrov et al., 2022). However, an important way of coping with grief is by talking, as it offers an opportunity to share feelings and experiences (Sikstrom et al., 2019).

According to the review, the shame caused by the cause of death prevented siblings from relying on their social networks. Even though attitudes towards substances may be more positive today, substance-related death is still a silent issue. Substance-related death is often preceded by a life situation that burdens loved ones as well (Mardani et al., 2023). Particularly, the self-accusations of the bereaved and thoughts of preventing a substance-related death can lead to prolonged grief (Mielenterveystalo, 2024).

The siblings’ efforts to distance themselves and set boundaries even before their sibling's substance-related death spoke of their self-valuing. The aim of setting such limits was to secure a quality of life and health. Weisner et al. (2010) have stated in their research that the healthcare costs of family members suffering from substance use disorders are significantly higher than those of sober people, and they have also been found to have more psychiatric and somatic diseases.

The results of this systematic literature review highlight that siblings experienced grief even before the death occurred. The sense of loss was present during the period of substance use, yet the death itself was often perceived as unexpected. This phenomenon of double loss warrants further research to understand how grief during substance use affects the grieving process and recovery after death.

The findings also strongly emphasize changes in family dynamics following the death. Many siblings minimized their own grief to avoid being seen as a burden. Further research is needed on the role of siblings within family dynamics both before and after the death of a sibling, including how they position themselves in the family and how this affects their grieving process.

### Reliability and Ethics of the Systematic Review

The reliability of this systematic review has been evaluated according to JBI systematic literature review evaluation criteria (Hotus, 2018b).

The inclusion criteria for the systematic review were defined precisely and compatible with the set research question. The selected articles met the defined inclusion criteria, and further articles were also selected for the review, which had simultaneously examined the perspective of other family members on the subject. In such cases, only the sibling's point of view was not completely distinguishable. The researchers decided to use those articles because there were only a few articles that included a sibling perspective alone. However, it is also acknowledged that a large number of the articles selected for the review arose from the same Norwegian project, which potentially weakens the reliability of the review.

The number of articles selected for the systematic review is small, but no further research information was found in the subject area. The systematic review includes only the articles identified with these search terms in three databases that addressed the research question. Consequently, some publications relevant to the topic may have been excluded.

The research articles were assessed for quality to be able to give weight to the results. Reliability is weakened by the fact that only one researcher carried out the quality assessment. However, the review is repeatable and the reliability of the review was critically evaluated at different stages of the research process (Petticrew & Roberts, 2008).

Inductive content analysis was used as the data analysis method in the initial search. The results of the updated search were analyzed deductively on the basis of the previous results framework.

Key results corresponding to the research question were collected from the selected studies and the information was combined into a whole (Hotus, 2018b). Little research has been carried out related to the subject area of the systematic review, which, given the statistical incidence of the research topic in Finland and most likely other countries, makes it necessary to strengthen the research evidence in the future (Hotus, 2018b).

## Conclusions

Based on the systematic review, the following conclusions are presented:

1. From the point of view of society and various support organizations, targeted support for siblings who have experienced the substance-related death of their own sibling may be completely lacking, and the responsibility for getting help is placed on the siblings themselves, at a time when this is most difficult.
2. Siblings’ grief may remain a secondary consideration after a substance-related death in the family because the sibling themself may feel that they are in some way responsible for others’ coping, or they may be given such responsibility.
3. The supportive attitude of other people and an open encounter is important in the sibling's survival process.
4. Stigma related to substance use stems from the prejudices and ignorance related to addiction diseases. Stigma affects not only a person with a substance use disorder, but also the loved ones of a person with a substance use disorder.

This systematic review shows that more research on siblings’ experiences is needed. Future research should also take into account young siblings, and in general the support that siblings receive and need after the death of their sibling.
